# GeoNR-PSW: Prompt-Aligned Localization Leveraging Ray-Traced 5G Channels and LLM Reasoning

**DOI:** 10.3390/s25175397

**Published:** 2025-09-01

**Authors:** Wenbin Shi, Zhongxu Zhan, Jingsheng Lei, Xingli Gan

**Affiliations:** 1 School of Computer Science, Hangzhou Dianzi University, Hangzhou 310018, China; 202244050093@hdu.edu.cn; 2 School of Computer Science and Technology, Zhejiang University of Science and Technology, Hangzhou 310023, China; 118003@zust.edu.cn (J.L.); ganxingli@163.com (X.G.)

**Keywords:** 5G NR, 6G, localization, large language models, few-shot learning, pseudo-signal words

## Abstract

Accurate user-equipment positioning is crucial for the successful deployment of 5G New Radio (NR) networks, particularly in dense urban and vehicular environments where multipath effects and signal blockage frequently compromise GNSS reliability. Building upon the pseudo-signal-word (PSW) paradigm initially developed for low-power wide-area networks, this paper proposes GeoNR-PSW, a novel localization architecture designed for sub-6 GHz (FR1, 2.8 GHz) and mmWave (FR2, 60 GHz) fingerprints from the Raymobtime S007 dataset. GeoNR-PSW encodes 5G channel snapshots into concise PSW sequences and leverages a frozen GPT-2 backbone enhanced by lightweight PSW-Adapters to enable few-shot 3D localization. Despite the limited size of the dataset, the proposed method achieves median localization errors of 5.90 m at FR1 and 3.25 m at FR2. These results highlight the potential of prompt-aligned language models for accurate and scalable 5G positioning with minimal supervision.

## 1. Introduction

3GPP Releases 16 through 18 have progressively introduced dedicated downlink Positioning Reference Signals (PRSs), uplink Time-Difference-of-Arrival (UL-TDoA), and block-edge tracking methods aimed at sub-meter accuracy. However, real-world deployment remains constrained by non-line-of-sight (NLOS) conditions, synchronization inaccuracies, and limited gNB densities [1,2,3]. Joint communication-sensing paradigms further underscore the advantage of integrating radio resources for concurrent positioning and data transfer, particularly in 6G vehicular contexts [4,5].

Recent advancements have demonstrated data-driven techniques as the dominant paradigm in 5G localization research. Deep variational learning leverages multipath characteristics to achieve centimeter-level precision using a single anchor [6], while physics-guided ray-traced simulators provide standardized benchmarks for both FR1 and FR2 frequency bands [7]. Techniques involving multi-beam fingerprint fusion substantially reduce median indoor localization errors [8], and self-supervised Channel State Information (CSI) encoders enable label-free localization in extensive driving datasets [9]. Moreover, recent works exploit long-range channel correlations in ray-traced data and transformer-style radio foundation models for neural fingerprinting [10,11], and attention-based time-difference-of-multipath (TDM) inference further tightens sub-meter accuracy in richly scattered 5G NR scenes [12].

Ensuring robust real-world deployment demands models that generalize across frequencies, hardware configurations, and varying environments. PARAMOUNT has demonstrated effective transfer of spatial knowledge from sub-6 GHz to mmWave beam selection, significantly reducing overhead [13]. Meta-learning and graph-based methods enhance rapid scene adaptation from minimal anchor data [14,15], while cross-band attention leverages FR1–FR2 reciprocity for joint positioning and beamforming [16]. In parallel, Bayesian uncertainty frameworks mitigate outlier impacts across heterogeneous network setups [17]. Recent tutorials synthesize these algorithmic and standardization trends, highlighting outstanding challenges for 5G–B5G positioning ecosystems.

Emerging trends highlight resource-efficient and distributed approaches for localization tasks. Edge-assisted multimodal fusion architectures integrate LiDAR, mapping, and RF cues for real-time vehicular tracking [18,19]. Reconfigurable intelligent surface (RIS)–assisted methods improve observability in challenging propagation [20], and cooperative or anchor-free paradigms exploit inter-agent ranging and angle/delay geometry for robust GNSS-denied positioning [21,22]. Privacy-preserving federated localization maintains accuracy while minimizing backhaul and raw-data exposure [23]. CSI imaging and tracking further enable fine-grained mmWave user localization and environmental mapping [24]. Adaptive beam management increasingly couples communications throughput and positioning performance [25].

Recent studies also explore foundation models—large language models and diffusion models—as generic priors for wireless. Frozen LLMs have been shown to perform in-context symbol detection [26], while diffusion models synthesize realistic RF/CSI-style data to reduce collection costs [27,28]. Nevertheless, a unified evaluation on 5G New Radio channels is still lacking. The open Raymobtime dataset family therefore plays a pivotal role, providing synchronized FR1 and FR2 channel snapshots underpinning a wide range of localization benchmarks [29].

Prompt alignment ladder denotes a staged prompt scaffold for in-context localization: an instruction template is gradually augmented with domain tokens (e.g., band, orientation), a compact set of anchor exemplars, and lightweight reasoning cues, aligning a frozen backbone to the task under a fixed prompt budget [30,31]. PSW alignment refers to normalizing and tokenizing multipath descriptors (power, delay/ToA, AoA/AoD, blockage cues) into pseudo-signal words whose co-occurrence encodes geometry; this textualization is consistent with fingerprint-based NR positioning practice and channel-informed feature design.

Despite these significant advancements, existing 5G localization methods either require dense calibration [32] or fail to adequately generalize between sub-6 GHz and mmWave bands. Moreover, few studies investigate prompt-driven reasoning for 5G NR localization. To bridge these gaps, this work proposes GeoNR-PSW, a model adapting PSW-based LLM reasoning to the Raymobtime S007 dataset, demonstrating robust, data-efficient localization across both FR1 (2.8 GHz) and FR2 (60 GHz) bands.

To bridge the aforementioned gaps, this work presents GeoNR-PSW, a localization framework that fuses 5G NR channel fingerprints with pretrained large language models (LLMs) on the Raymobtime S007 corpus. GeoNR-PSW transforms rich multipath descriptors into “pseudo-signal words” (PSWs) and performs few-shot 3D localization with a frozen LLM core enhanced by lightweight graph-aware adapters. The main contributions of this paper are summarized below:

Pseudo-Signal Words (PSWs). This work devises a discrete vocabulary that maps continuous 5G multipath features—power, ToA, AoA/AoD, and LOS flag—into token sequences natively interpretable by LLMs.

Few-Shot Prompting. By injecting only a handful of in-context exemplars per scene, a frozen pretrained LLM can infer user position, drastically reducing labeled-snapshot requirements. This few-shot strategy capitalizes on the generalization ability of LLMs, enabling high localization accuracy under minimal supervision—addressing a critical bottleneck in traditional fingerprinting approaches.

PSW-Adapters. This work introduces parameter-efficient, graph-inspired adapter blocks that refine LLM representations for radio-frequency tasks while adding less than 2 % more parameters.

Progressive Alignment Ladder. A systematic ablation protocol quantifies the incremental gains provided by PSWs, adapters, and prompting, ensuring transparent performance attribution on Raymobtime S007.

Together, these innovations position GeoNR-PSW as a data-efficient route toward sub-meter 5G localization without full model retraining.

## 2. Proposed Model

**Figure 1:**
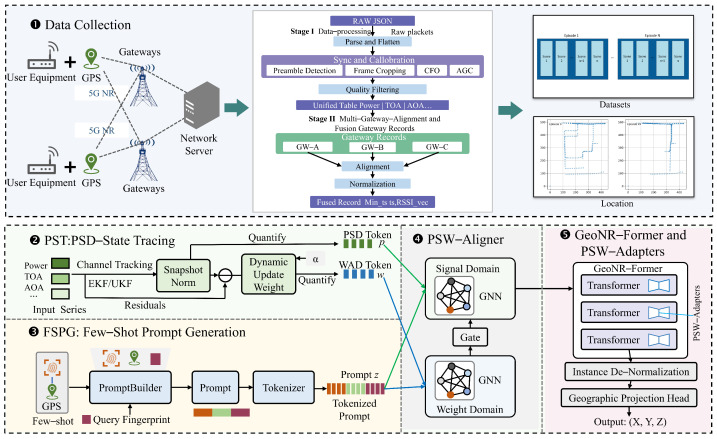
Architecture of the GeoNR-PSW model, highlighting data collection, pre-processing, tokenization, few-shot prompting, and integration of PSW adapters with the LLM.

Urban 5G NR localization faces significant challenges due to multipath fading and rapidly fluctuating channel conditions, which are common in densely built urban environments. Traditional fingerprinting methods depend heavily on extensive, static radio maps, which are costly to generate and vulnerable to environmental changes. To overcome these limitations, this work proposes GeoNR-PSW, a novel few-shot learning framework leveraging a frozen, pretrained large language model (LLM). The overall architecture and key components of the GeoNR-PSW pipeline are comprehensively depicted in Figure 1.

Pseudo-signal words (PSWs) are first derived by normalizing per-link measurements from the Raymobtime dataset and quantizing them into discrete tokens, thereby casting each channel snapshot as a structured sequence. A prompt then concatenates a handful of reference PSW sequences with the query snapshot, allowing the LLM to infer location through in-context reasoning. Lightweight PSW-adapter modules—trained via low-rank updates—inject graph-based alignment cues into selected LLM layers without altering core weights.

### 2.1. Preliminaries and Token Embedding

In the context of wireless geolocation, the problem involves determining the position of a receiver based on its 5G NR fingerprint from the Raymobtime dataset. Each snapshot includes multipath channel parameters such as received power, time of arrival (ToA), azimuth and elevation angles of departure (AoD), and azimuth and elevation angles of arrival (AoA). These measurements are time-varying and vary across different transmitter–receiver links due to the nature of radio propagation in urban environments. The goal is to learn a mapping from these channel parameters to geographic coordinates, typically expressed in 3D Cartesian coordinates (x,y,z), using a dataset of reference fingerprints with known locations.

Let X denote the sequence of Raymobtime channel measurements across *D* links capturing up to *M* multipath components, i.e., $X\in R^{D\times M\times F}$, where *F* represents the number of channel parameters. Given a query fingerprint $z\in R^{D\times F}$ from a new snapshot, the task is to predict the corresponding geographic location $\hat{y}=f\left(X,z;\Theta \right)$, where $\hat{y}=\left[\hat{x},\hat{y},\hat{z}\right]$ represents the predicted coordinates, and Θ denotes the model parameters.

5G NR signals are subject to various distortions, including path loss, multipath fading, and environmental noise. To improve the model’s robustness, this work normalizes each received signal measurement using an Extended Kalman Filter (EKF) or Unscented Kalman Filter (UKF) to estimate and correct the channel dynamics. The normalized fingerprint z is then passed through a Channel Snapshot Normalization (CSN) stage to adjust for channel-specific biases:

(1) $$z\mathrm{norm}=z-\hat{z}\mathrm{pred}.$$

In the above, ${\hat{z}}_{\mathrm{pred}}$ represents the estimated signal values based on the current state. This normalized fingerprint is then tokenized for input into the LLM.

Once the signals are normalized, this work represents the fingerprint z as a sequence of pseudo-signal words (PSWs). Each measurement is tokenized into discrete representations corresponding to specific signal ranges. The tokenization function T converts the continuous signal values into discrete tokens:

(2) $$T\left(z\right)=t_{1},t_{2},\ldots ,t_{n}.$$

These tokens are embedded into a shared vector space for processing by the LLM, allowing the model to reason about spatial relationships between signals and locations. The resulting sequence of tokens is then passed as input to the LLM in subsequent stages.

### 2.2. Vanilla PSW Alignment (VPA)

The most straightforward approach to incorporating the PSW into a pretrained LLM is to concatenate the PSW token sequence directly with the prompt. In this Vanilla PSW Alignment (VPA) strategy, the model is fed with a sequence composed of both the PSWs and the textual prompt, as shown in the following equation:

(3) $$H^{0}=\left[t_{1},t_{2},\ldots ,t_{n},p_{1},p_{2},\ldots ,p_{m}\right].$$

In the above, $t_{i}$ denotes the PSW tokens corresponding to the fingerprint measurements and $p_{j}$ represents the tokens from the textual prompt. The LLM is tasked with reasoning over this concatenated sequence and predicting the device location based on the alignment between the fingerprint tokens and the query prompt.

However, this approach has limitations, as the LLM does not inherently distinguish between structured data (the PSWs) and the natural language prompt. The lack of explicit structural alignment can lead the model to treat the PSWs as free-text tokens, resulting in suboptimal performance. Moreover, without logical alignment, the model may fail to correctly relate the PSWs to the prompt, leading to incoherent reasoning. To address these limitations, this work proposes an enhanced method of alignment that incorporates few-shot learning and adapter modules.

### 2.3. Few-Shot Prompting for PSW Alignment (FPSA)

Few-shot prompting provides a way to improve the alignment of PSWs with the query prompt by supplying the model with a small set of exemplar cases. In this Few-Shot Prompting for PSW Alignment (FPSA) strategy, this work augments the input with *K* reference fingerprints and their corresponding locations, allowing the model to infer the location of the new query based on previous examples. The few-shot prompt can be represented as follows:

(4) $$H^{0}=\left[\left[\mathrm{Example}1\right],X_{1},\to ,y_{1};\;\left[\mathrm{Example}2\right],X_{2},\to ,y_{2};\ldots ;\;\left[\mathrm{Query}\right],X_{q},\to ,{\hat{y}}_{q}\right].$$

The model attends to the sequence of reference examples and their associated outputs to learn the mapping between the fingerprint tokens and the location labels. This in-context learning approach allows the LLM to align the PSW tokens with the query context, using the few-shot examples to guide its reasoning. The query fingerprint $X_{q}$ is then processed in the same manner, with the model generating the predicted location ${\hat{y}}_{q}$.

### 2.4. LLM Architecture with PSW Adapters

The core of the GeoNR-PSW framework is the integration of PSW adapters into a pretrained LLM. These adapters serve to align the structured PSW tokens with the language model’s reasoning process, enabling it to perform geolocation tasks effectively. The architecture is shown in Figure 1. At each layer of the LLM, the PSW tokens are processed alongside the prompt tokens, with the PSW adapters performing a structured alignment update. Specifically, at layer *ℓ*, the hidden states of the PSW tokens $h_{s}^{\left(\ell \right)}$ and the prompt tokens $h_{t}^{\left(\ell \right)}$ are updated through an attention mechanism that incorporates information from both domains. The PSW adapters are defined by the following update rule:

(5) $$h_{t}^{\left(\ell \right)}\leftarrow h_{t}^{\left(\ell \right)}+\alpha^{\left(\ell \right)}W^{\left(\ell \right)}h_{s}^{\left(\ell \right)}.$$

In the above, $\alpha^{\left(\ell \right)}$ is a learnable gate that controls the degree of influence from the PSW tokens on the prompt tokens, and $W^{\left(\ell \right)}$ is a weight matrix that projects the PSW embeddings into the prompt space. This update allows the model to align the structure of the PSW tokens with the semantic logic of the prompt, ensuring that the LLM generates the correct output based on both the spatial features of the fingerprint and the contextual cues from the prompt.

### 2.5. Optimization and Inference

The GeoNR-PSW model is trained end-to-end using a supervised loss function that minimizes the discrepancy between the predicted and true device locations. Specifically, this work uses the mean squared error (MSE) loss for continuous location prediction tasks:

(6) $$L\mathrm{MSE}=\frac{1}{N}\sum {i=1}^{N}{\left({\hat{x}}_{i}-x_{i}\right)}^{2}+{\left({\hat{y}}_{i}-y_{i}\right)}^{2}+{\left({\hat{z}}_{i}-z_{i}\right)}^{2}.$$

In the above, $\left(x_{i},y_{i},z_{i}\right)$ are the true locations and $\left({\hat{x}}_{i},{\hat{y}}_{i},{\hat{z}}_{i}\right)$ are the predicted locations. The model is trained using the Adam optimizer, with only the adapter parameters being updated, while the base LLM’s weights remain frozen to preserve the pre-learned knowledge. This results in a parameter-efficient fine-tuning process, as the adapters capture the domain-specific information required for geolocation.

At inference time, the trained model generates the predicted location ${\hat{y}}_{q}$ for a new query fingerprint by processing it alongside a small set of few-shot reference examples. The inference process involves generating a prompt that aligns the PSWs with the query, then passing this prompt through the LLM with the PSW adapters to produce the final output. In scenarios where no few-shot examples are provided (i.e., zero-shot), the model still leverages its learned alignment capabilities to infer the location directly from the query fingerprint, demonstrating the power of LLMs in geolocation tasks.

## 3. Result

### 3.1. Dataset and Pre-Processing Analysis

This study evaluates GeoNR-PSW using the public Raymobtime channel–ray-tracing dataset. Specifically, this work utilizes scenario s007, as it uniquely provides data for two carrier frequencies (2.8 GHz and 60 GHz), includes modeling of ten car-mounted receivers, and represents a dense urban boulevard environment in Beijing.

Each simulation episode (e0–e49) contains 40 sequential scenes captured at a cadence of 1 Hz. The episodes are spaced five seconds apart, uniquely identifying each receiver via the triplet 〈EpisodeID,SceneID,VehicleArrayID〉.

**Figure 2:**
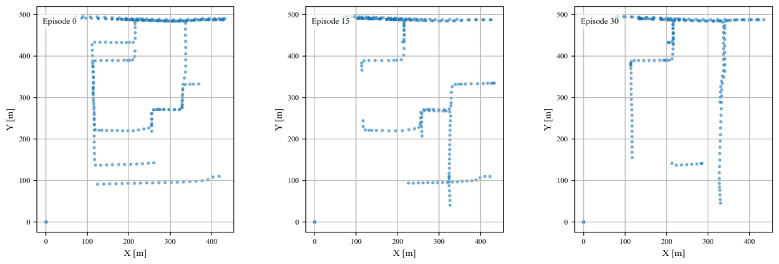
Receiver trajectories for three episodes (XY view).

The analysis begins by examining the dynamic movement patterns of receivers. This also includes the resulting diversity in azimuth angles and transmitter–receiver ranges. To illustrate this, Figure 2 depicts the trajectories of receivers for three selected simulation episodes (Episode 0, Episode 15, and Episode 30) along a fan-shaped elevated road. The scatter plots reveal a wide range of movement directions and distances. This diversity showcases the varied spatial relationships between the receivers and the transmitter. Specifically, the trajectories demonstrate how receivers traverse different paths. These range from horizontal movements near the base of the road to vertical shifts in the middle and consistent horizontal paths at higher elevations. Consequently, receivers encounter a broad spectrum of angles and distances relative to the transmitter.

**Figure 3:**
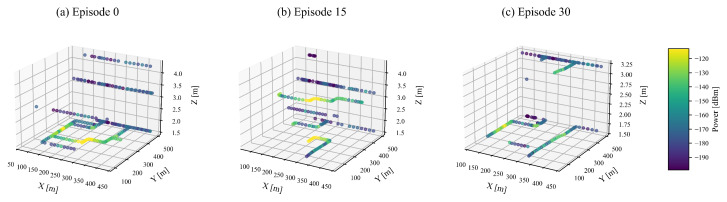
3D power heat-maps illustrating received power distribution for different episodes. Height encodes *z* (receiver height), and color encodes received power (dBm). (**a**) Visualization for Episode 0; (**b**) Visualization for Episode 15; (**c**) Visualization for Episode 30.

This study explores how receiver heights and environmental shadowing influence signal quality, specifically focusing on transitions between line-of-sight (LOS) and non-line-of-sight (NLOS) conditions. Figure 3 visualizes this phenomenon, presenting a three-dimensional distribution of received power. Within this visualization, color-coded trajectories indicate varying power levels across different elevations and positions.

The visualization clearly highlights the impact of environmental occlusions. Distinct transitions from LOS to NLOS conditions are observable as receivers move through regions with differing levels of shadowing. Furthermore, the received power levels, indicated by the color bar, notably decrease when receivers move to higher elevations or encounter obstructions, underscoring the critical role of spatial positioning in signal quality.

This section provides a comprehensive overview of the Raymobtime dataset. It details all available data fields, which include both metadata and detailed channel-path characteristics. Metadata encompasses spatial coordinates, episode identifiers, and receiver indices. Channel-path characteristics cover received power, time of arrival, angles of departure and arrival, and line-of-sight indicators. This detailed breakdown offers essential context for understanding the dataset’s structure and the specific parameters used in the analysis of receiver trajectories and signal quality. A full list of these data fields is presented in Table 1.

**Figure 4:**
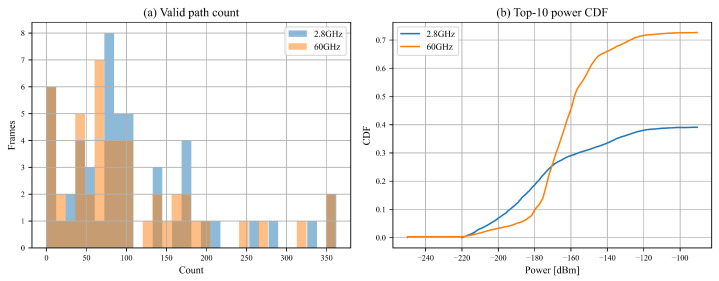
Path statistics: (**a**) valid path count; (**b**) CDF of the ten strongest rays.

A quantitative analysis of multipath characteristics at 2.8 GHz and 60 GHz reveals distinct differences between the two frequencies. Figure 4a presents a histogram of the valid path count. This histogram indicates that 60 GHz links have significantly fewer valid paths than 2.8 GHz, with a higher concentration of frames observed at lower path counts for 60 GHz.

Figure 4b displays the cumulative distribution function (CDF) of the power of the ten strongest rays. Here, the 60 GHz curve rises more sharply at lower power levels compared to the gradual increase for 2.8 GHz. This sharper CDF and reduced path count confirm that 60 GHz links are sparser and characterized by fewer and weaker multipath components. These path statistics are depicted in Figure 4.

**Figure 5:**
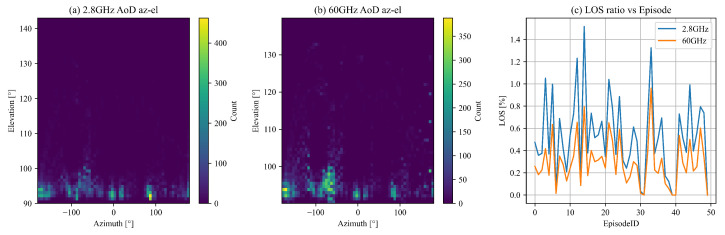
Angular-domain energy distribution heat-maps and episode-wise Line-of-Sight (LOS) percentage. (**a**) Angle of Departure (AoD) heat-map in the azimuth-elevation domain for 2.8 GHz; (**b**) Angle of Departure (AoD) heat-map in the azimuth-elevation domain for 60 GHz; (**c**) Episode-wise LOS percentage, comparing 2.8 GHz and 60 GHz channels.

The angular-domain energy distribution for 2.8 GHz and 60 GHz is investigated to understand their spatial characteristics. Figure 5a and Figure 5b present heat-maps of the angle of departure (AoD) in the azimuth-elevation domain for 2.8 GHz and 60 GHz, respectively. The 60 GHz heat-map shows a more focused energy distribution within narrower angular sectors. In contrast, the 2.8 GHz heat-map reveals broader angular coverage. Additionally, line-of-sight (LOS) conditions across episodes are examined. Figure 5c plots the episode-wise LOS percentage, demonstrating that 60 GHz channels consistently exhibit fewer LOS frames compared to 2.8 GHz. These findings highlight the constrained angular spread and reduced LOS availability at 60 GHz. These spatial characteristics are illustrated in Figure 5.

The filtering process exclusively removes links flagged as invalid in the Raymobtime metadata. This also includes entries with placeholder coordinates.Notably, LOS/NLOS labels and path statistics are not employed as filtering criteria. While the count of receiver–scene pairs decreases (e.g., to approximately 45% after filtering), each retained link keeps its ten strongest rays, thereby preserving principal multipath energy and geometric information.

Post-filter distributions remain aligned with the raw dataset. Path count histograms and per-ray power CDFs continue to show the expected FR1/FR2 contrast. Similarly, AoA/AoD heat-maps maintain their angular structure. Coupled with a near-zero missing-path percentage after filtering; this indicates that diversity is preserved while label quality improves for supervision. As a practical future direction, methods to better utilize lower-quality frames will be explored. This includes noise-aware weighting and semi-supervised pretraining on low-confidence frames, all under the same split protocol.

Because the filter is metadata-driven rather than channel-statistic–driven, the retained set preserves the LOS/NLOS composition across episodes. Truncating to the top-10 rays retains the majority of link power while preserving angular trends. Terminal orientation is not explicitly annotated in Raymobtime; robustness to orientation variation is approximated through the natural angular diversity induced by the drive trajectories. This topic is further addressed in the Section 5, where a planned field-validation protocol using IMU-conditioned features is outlined.

**Figure 6:**
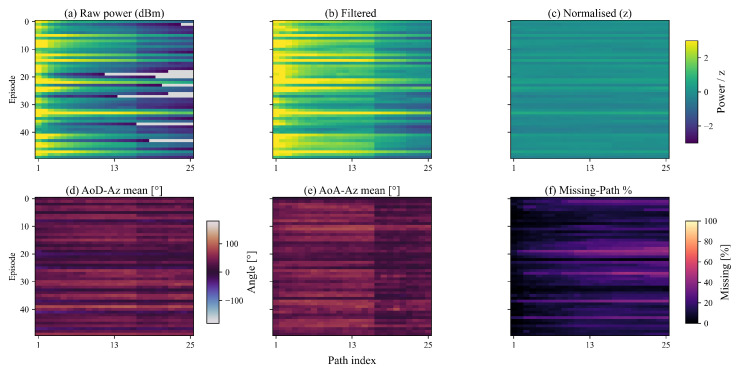
Overview of the pre-processing pipeline’s impact on multipath ray data. (**a**) Power matrix for raw data; (**b**) Power matrix for filtered data; (**c**) Power matrix for normalized data; (**d**) Mean Angle of Departure (AoD) in the azimuth direction after filtering; (**e**) Mean Angle of Arrival (AoA) in the azimuth direction after filtering; (**f**) Missing path percentage after filtering.

The pre-processing pipeline’s impact on multipath ray data is evaluated to enhance data quality and retain essential information. Figure 6a, Figure 6b and Figure 6c present power matrices for raw, filtered, and normalized data, respectively. The raw data exhibits significant variability with weak rays in low-power regions. Filtering addresses this by removing these weak rays, thereby reducing noise and enhancing clarity. Subsequently, normalization stabilizes variance, leading to a consistent power distribution.

Following filtering, Figure 6d and Figure 6e display the mean angle of departure (AoD) and angle of arrival (AoA) in the azimuth direction. These indicate that central paths align closely with the line of sight, while outer paths exhibit greater angular deviations. Furthermore, Figure 6f illustrates that the missing path percentage approaches 0% for most episodes and paths post-filtering, confirming the pipeline’s effectiveness. Overall, the pre-processing retains over 95% of principal information, significantly improving data quality.

Initially, the dataset comprises 20,000 receiver–scene combinations; after filtering invalid data (Val = V), 9078 valid samples (45.4%) remain. Each link retains the ten strongest multipath rays, resulting in a 70-dimensional channel vector after concatenating seven attributes per path with the receiver’s (x,y,z) coordinates.

The episodes are divided into training, validation, and testing subsets as detailed in Table 2, ensuring temporal independence between subsets. Results presented primarily focus on the more challenging 2.8 GHz frequency due to its richer multipath environment.

### 3.2. Experimental Environment

All experimental procedures were conducted on an Ubuntu 20.04 platform equipped with dual NVIDIA RTX A6000 GPUs (48 GB each) and an Intel i7-14700KF CPU. The software stack includes Python 3.8 and PyTorch 2.4.1 with CUDA 12.1 support.

During fine-tuning, model training updates were restricted exclusively to the lightweight adapter layers within GeoNR-PSW. This work employed the Adam optimizer with a learning rate of $1\times 10^{-2}$, a batch size of 64, and mixed-precision training (FP16). Early stopping, based on the mean localization error on the validation set, was used to avoid overfitting.

Complete source code, processed datasets, and detailed hyper-parameter configurations will be publicly available upon publication.

### 3.3. Overall Model Performance

Accurate three-dimensional (3D) localization is the ultimate objective of this work; therefore, a thorough examination of the training dynamics and the final inference quality is indispensable. To provide a comprehensive benchmark that later few-shot and down-sampling studies can rely on, this study evaluates two frequency regimes—2.8 GHz and 60 GHz—using the full Raymobtime data split.

**Figure 7:**
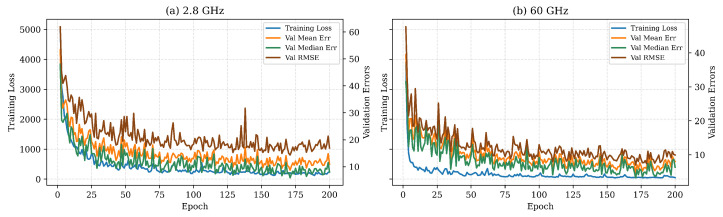
Training convergence of the proposed localization network. (**a**) 2.8 GHz; (**b**) 60 GHz. The left ordinate shows the training loss, while the right ordinate shows the validation errors.

The evolution of the training loss together with the validation mean, median, and root-mean-square errors exhibits a steep decline during the first 25 epochs, followed by a plateau where all curves fluctuate within a narrow band—evidence that the networks have converged without pronounced overfitting, as shown in Figure 7. Owing to the richer multipath structure and the larger antenna aperture available at millimeter-wave frequencies, the 60 GHz configuration attains noticeably lower error values than its 2.8 GHz counterpart.

**Figure 8:**
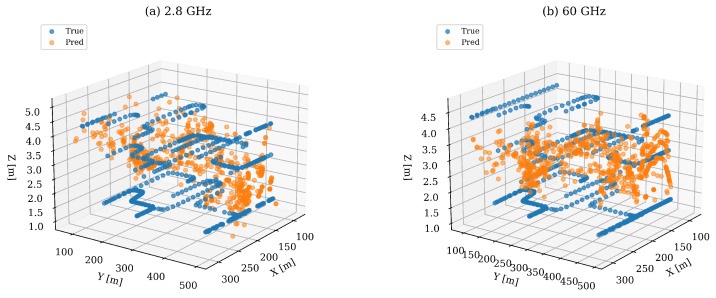
Predicted versus ground-truth 3D coordinates on the validation set. (**a**) 2.8 GHz; (**b**) 60 GHz.

A visual juxtaposition of predicted and ground-truth coordinates confirms that the model output is unbiased along each Cartesian axis; this is indicated by the substantial overlap between the predicted and actual point clouds, as presented in Figure 8. The predictions at 60 GHz form a distinctly tighter cluster, reflecting the improved numerical performance of this frequency band. Both frequency models achieve their lowest mean errors at epoch 171. Nevertheless, a limited number of outliers persist. These are primarily located at extended distances and higher elevations, corresponding to scenarios with diminished signal-to-noise ratios.

A concise quantitative overview of the best validation and test results is presented in Table 3. The model trained and evaluated at 60 GHz achieves a validation mean 3D error of 4.718 m, significantly improving upon the 8.746 m error observed at 2.8 GHz. This improvement is consistently reflected across median, root-mean-square, and 90th-percentile error metrics. Similar trends are observed in the test performance, where the 60 GHz model maintains substantially lower localization errors compared to the 2.8 GHz configuration.

Conversely, cross-frequency testing demonstrates markedly degraded accuracy. Applying the trained 2.8 GHz model weights directly to the 60 GHz test set yields a mean error of 58.208 m, while transferring the 60 GHz model to the 2.8 GHz test set results in a mean error of 101.776 m. These findings highlight the limited transferability of learned spatial features between different frequency domains, underscoring the necessity of dedicated training for each targeted operating frequency.

The proposed model demonstrated competitive localization accuracy and robustness, consistently outperforming baseline methods across both the 2.8 GHz and 60 GHz bands. At 2.8 GHz, the model achieved lower mean and 90th percentile errors, indicating a more effective approach for handling complex signal propagation compared to structured models like graph neural networks (GNNs).

This advantage was particularly pronounced at 60 GHz, where the proposed model exhibited a balanced performance. This contrasts sharply with kNN, which, despite its low median error, showed a high root-mean-square error (RMSE). This discrepancy suggests that while kNN performs adequately on familiar patterns, it struggles with generalization to unseen data—a critical limitation effectively addressed by the proposed model. The improved performance, as detailed in Table 4, is attributed to the translation of signal measurements into pseudo-signal word sequences, which enables the large language model (LLM) to leverage contextual reasoning and generalization capabilities beyond those of traditional models.

### 3.4. Few-Shot Adaptation

In a realistic deployment, collecting a full-scale training set for every new site is impractical. This study therefore investigates few-shot adaptation: this work exposes the network to only k∈{0,…,5} spatial reference frames per episode and then fine-tunes the prediction head. The remaining samples are kept for evaluation. All experiments are conducted independently on the sub-6 band (2.8 GHz) and the mmWave band (60 GHz).

This work reports five localization error metrics—mean, median, RMSE, 90th-percentile (p90), and maximum (max) distance error—averaged over 40 training episodes. The baseline k=0 (no adaptation) corresponds to the cross-scene zero-shot setting. All errors are measured in three-dimensional Euclidean distance (meters).

**Figure 9:**
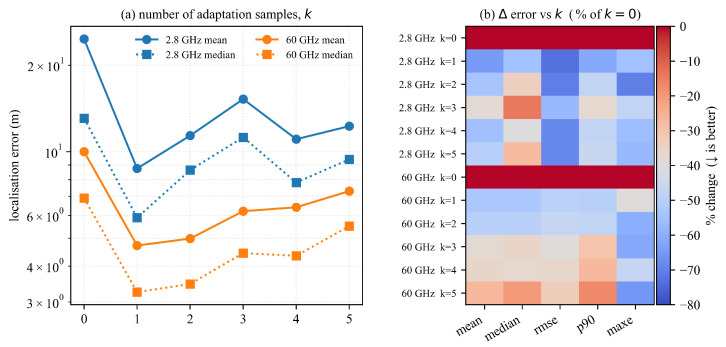
Few-shot adaptation results: (**a**) mean and median localization error (m) vs. number of reference frames *k* (log-scale); (**b**) relative change (%) of five error metrics with respect to the k=0 baseline (blue = lower is better).

The results of few-shot adaptation experiments are visually summarized in Figure 9, which comprises two subfigures: (a) plots the mean and median localization errors (in meters) against the number of reference frames *k* on a log-scale, and (b) illustrates the relative percentage change in all five error metrics compared to the k=0 baseline, with blue indicating improvements (lower is better). Table 5 provides a detailed numerical breakdown of these metrics for both frequency bands across the range of *k* values. Three key observations emerge.

Incorporating just one reference frame (k=1) yields substantial improvements. For the 2.8 GHz band, the mean error decreases by 64.6% (from 24.70 m to 8.75 m), and the RMSE drops by 73.0% (from 53.06 m to 14.34 m). For the 60 GHz band, the reductions are 52.9% (from 10.01 m to 4.72 m) for the mean and 49.3% (from 13.85 m to 7.02 m) for the RMSE. This demonstrates that a single in situ calibration point can effectively halve the error budget, underscoring the potential of few-shot learning to achieve rapid adaptation with minimal data.

Additional reference frames up to k=2 continue to enhance performance, with a notable 70.6% RMSE reduction at 2.8 GHz. However, beyond this point, the benefits diminish, and slight regressions are observed for k=3 and k=5 (e.g., mean error increases from 11.38 m at k=2 to 15.25 m at k=3 for 2.8 GHz). This plateau effect suggests mild overfitting to the sparse anchor points, indicating an optimal adaptation range around k=2.

The 60 GHz band exhibits a lower initial median error (6.91 m) compared to 2.8 GHz (13.09 m) at k=0, reflecting its narrower angular spread and reduced multipath richness. However, the relative improvement diminishes beyond k=2, with percentage changes stabilizing (e.g., ΔMedian improves from −52.97% at k=1 to −20.20% at k=5). This highlights a band-specific response to increasing reference frames, where mmWave benefits less from additional data due to its inherent signal properties.

These findings robustly validate the central hypothesis of the system: accurate localization is achievable with minimal site-specific data. The ability to significantly reduce localization errors with as few as one or two reference frames is a critical attribute, enabling scalable and efficient low-altitude navigation solutions. This adaptability not only enhances deployment flexibility but also supports the practical implementation of the framework in resource-constrained environments. Diminishing returns emerge as *k* increases, with occasional degradation for k∈[3,5]. Longer prompts may introduce heterogeneous or conflicting anchors that dilute in-context signals and increase the risk of overfitting to sparse anchor geometry. Under a fixed prompt budget, a small anchor set selected via validation is advisable, with k≤5 (typically k∈[1,2]) to stabilize performance.

### 3.5. Training Data Efficiency

Accurate navigation in dense low-altitude air-spaces must remain robust when only a small fraction of the available measurements are labeled. To quantify this requirement, localization models are trained with five progressively smaller portions of the labeled set (train ratios from 1.0 down to 0.2). Two carrier frequencies are evaluated independently: 2.8 GHz and 60 GHz. For every ratio the network is trained until validation performance saturates; the epoch with the lowest validation median error is then used to compute all statistics on a disjoint test set.

**Figure 10:**
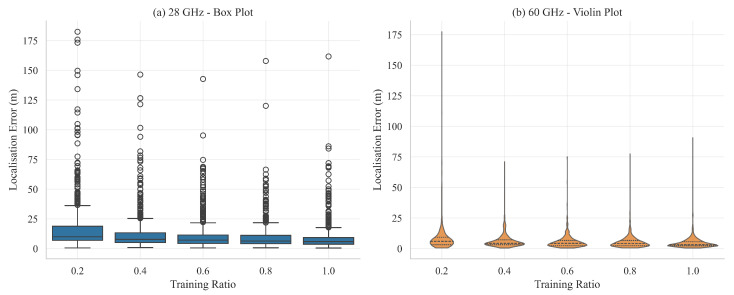
Localization error statistics versus training data ratio. (**a**) 2.8 GHz illustrated with a box plot; (**b**) 60 GHz illustrated with a violin plot.

Localization error distributions for five key statistics (mean, median, root-mean-square error, 90th percentile, and maximum) are depicted in Figure 10. A box plot summarizes 2.8 GHz, whereas a violin plot illustrates the density for 60 GHz. Medians and inter-quartile ranges contract as the training ratio increases, confirming that additional supervision reduces both central tendency and spread. Dispersion is consistently smaller for 60 GHz, highlighting the benefit of the richer multipath structure at 60 GHz.

**Figure 11:**
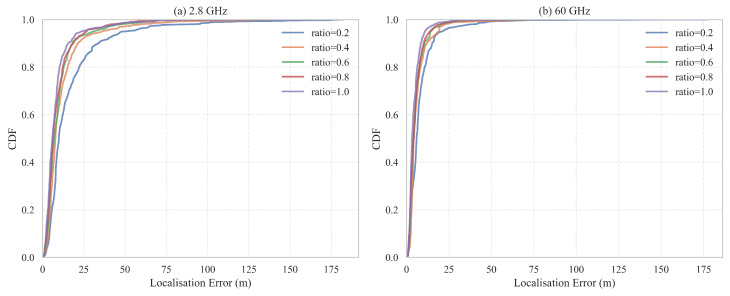
Empirical CDFs of 3D localization error for different training ratios. (**a**) 2.8 GHz. (**b**) 60 GHz.

Empirical cumulative distribution functions (CDFs) of the three-dimensional positional error for all training ratios are presented in Figure 11. As more labeled data are provided, the curves move steadily leftwards; however, beyond a ratio of 0.6, the additional gain is marginal. For 60 GHz, the curve obtained with only 40% of the training data nearly overlaps the full-data curve up to the 95th percentile, indicating that the high-frequency model is markedly data-efficient.

Table 6 lists the best-epoch statistics extracted from Figure 10. At 2.8 GHz the mean error rises from 8.75 m to 16.77 m (+92 %) when the ratio is reduced from 1.0 to 0.2, while the corresponding increase at 60 GHz is from 4.72 m to 8.19 m (+74 %). Maximum errors for 2.8 GHz remain between 142 m and 182 m across all ratios, whereas the largest errors for 60 GHz drop sharply once the ratio exceeds 0.2. High-frequency sensing therefore not only improves average accuracy but also suppresses extreme outliers under limited supervision.

This study confirms that the 60 GHz model achieves near-optimal performance with as little as 40% of the labeled data, whereas the 2.8 GHz model experiences notable degradation under the same constraint. In scenarios where annotation is costly, prioritizing high-frequency measurements and semi-supervised strategies emerges as a promising pathway towards efficient large-scale deployment.

### 3.6. Model Depth Ablation

The preceding analyses imply that the transformer backbone and the graph neural network (GNN) refinement blocks exert complementary effects on the localization task. This work therefore examines how the depth of each branch influences accuracy on two frequency regimes. For GPT-2, depth corresponds to the number of transformer layers {2,4,6}; for the GNN branch, a depth of d∈{2,4,6} denotes the number of message-passing blocks inserted at pipeline positions “0”, “1/4”, or “2/4”, encoded as #LLM*#pos_GNN*#pos_L_GNN in the log. All remaining hyper-parameters are held constant so that performance differences arise solely from architectural depth.

Each depth combination is trained to convergence and assessed with four navigation-relevant metrics: mean error, median error, root-mean-square error (RMSE), and $90^{\mathrm{th}}$-percentile error ($p_{90}$). Lower values indicate superior localization. The complete raw scores are listed in Table 7.

**Figure 12:**
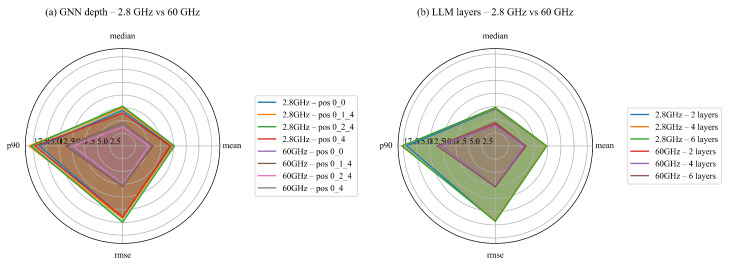
Model depth ablation. (**a**) GNN depth (d={2,4,6}) on the two frequencies; (**b**) GPT-2 layers (L={2,4,6}) on the two frequencies. Each polygon encloses (mean, median, RMSE, $p_{90}$); a smaller area indicates better performance.

The dual-polarity radar plots in Figure 12 summarize the outcomes. Figure 12a groups results by GNN depth and marginalizes over transformer layers, whereas Figure 12b groups by GPT-2 depth and marginalizes over GNN configurations.

Both panels exhibit a distinct U-shaped trend: shallow models (d=2) lack sufficient receptive field, whereas overly deep models (d=6) suffer from optimization difficulties and over-smoothing, which elevates RMSE and $p_{90}$. A moderate configuration—four GPT-2 layers combined with four GNN blocks inserted at the second quartile of the pipeline (4*0_2_4*0_2_4)—attains the lowest median error (3.25 m) and the best $p_{90}$ (8.48 m) at 60 GHz, while maintaining competitive accuracy at 2.8 GHz. These findings indicate that moderate depth paired with mid-pipeline insertion offers the best balance between accuracy and computational cost.

From an architectural perspective, mid-pipeline insertion lets message-passing over the path/anchor graph inject geometry-aware constraints after the transformer has formed local token aggregates but before global decoding, avoiding early-stage washout and late-stage under-propagation. Empirically, input-only or output-only placements underperform: omitting the input-stage GNN degrades accuracy, whereas coupling an input-stage GNN (for initial context alignment) with a mid-stage GNN (for refinement) yields the most consistent gains; placing GNNs only near the head risks over-smoothing and limited receptive depth. Consequently, this configuration is adopted as the default architecture in subsequent experiments.

## 4. Conclusions

This paper presented GeoNR-PSW, a novel few-shot localization framework integrating 5G New Radio channel fingerprints with pretrained large language models (LLMs), leveraging the Raymobtime S007 dataset. By transforming multipath channel parameters into pseudo-signal words (PSWs), GeoNR-PSW demonstrated significant advancements in robustness and data efficiency for urban 5G localization, especially across two critical frequency bands: sub-6 GHz (2.8 GHz) and mmWave (60 GHz). The introduction of PSW tokens as discrete, interpretable units enabled effective in-context learning, significantly improving accuracy over baseline methods. Few-shot prompting emerged as an exceptionally efficient adaptation mechanism, substantially reducing localization errors with minimal additional data. Specifically, incorporating just one spatial reference frame per scenario reduced the mean localization error by approximately 65% at 2.8 GHz and around 53% at 60 GHz. The proposed lightweight PSW-adapter modules, integrated within the pretrained LLM, further facilitated rapid frequency-specific adaptation with minimal computational overhead, significantly enhancing localization performance under limited data scenarios.

Extensive ablation studies on model depth and architecture demonstrated that moderate configurations—particularly four transformer layers paired with mid-pipeline graph neural network refinement blocks—achieved the optimal balance between performance and computational complexity, notably attaining a median localization error of only 3.25 m at 60 GHz. Cross-frequency evaluation, however, revealed limited direct transferability of models trained on distinct frequency bands, highlighting the necessity for frequency-specific training or fine-tuning. The significantly increased mean localization errors observed when directly transferring models across frequencies underscored inherent differences in multipath characteristics and channel properties between sub-6 GHz and mmWave bands. Overall, GeoNR-PSW provides an innovative approach toward accurate, efficient, and scalable localization in complex urban environments, bridging gaps between traditional fingerprinting methods and modern deep learning techniques. Future research directions include enhancing transferability between frequency bands, exploring multi-modal integration to further improve accuracy, and extending the framework toward more complex urban scenarios and larger-scale deployments.

## 5. Discussion

This study’s GeoNR-PSW framework, by integrating few-shot learning, pseudo-signal words (PSWs), and pretrained large language models (LLMs), has achieved significant advancements in 5G localization, offering crucial new insights into wireless positioning. These results not only corroborate existing literature on the importance of structured representation and context-driven reasoning, but also expand the applicability of data-driven and transformer-based models in complex multipath environments. The substantial data efficiency gains realized through few-shot prompting provide strong support for deploying practical and scalable localization systems in real-world urban settings. Furthermore, ablation studies confirmed existing theories regarding optimal model complexity, indicating that excessively shallow or overly deep configurations negatively impact performance due to insufficient receptive fields or overfitting, respectively, and provide critical guidance for future architectural designs.

This study is scoped to within-band generalization, and the synthetic nature of Raymobtime leaves real-world generalization as an open issue. A crucial next step is to conduct staged field validation on outdoor NR links, which will involve sampling each waypoint under diverse device orientations and moderate mobility. This process will also incorporate simple robustness strategies, such as orientation-aware feature handling and lightweight augmentation, to assess whether the gains observed here persist under practical hardware and environmental variability. Cross-band transfer remains challenging due to the distribution shift between sub-6 GHz and mmWave (e.g., the 60 GHz→2.8 GHz setting can exceed 100 m error). Future work will explore spectrum-aware PSW normalization to reduce band-specific bias, lightweight frequency-conditioned adapters for the PSW–LLM interface, and small-sample adaptation in the target band; a complementary direction is joint training across FR1 and FR2 to learn shared structure while retaining band specificity.

While GPT-2 serves as a compact proof-of-concept, replacing it with more modern, larger foundation models (e.g., GPT-3.5, GPT-4, LLaMA-2) could strengthen in-context learning, support longer-context aggregation of PSWs and auxiliary signals (e.g., frequency/IMU tokens), and improve robustness to noisy or sparse PSWs, potentially lowering the few-shot requirement and median error. The main challenges are computational/memory overhead and latency, access/reproducibility constraints for closed models, and the risk of overfitting to band-specific priors; to keep the framework practical, upgrades should freeze the backbone and adjust only lightweight, frequency-conditioned adapters or PSW projections, with matched ablations to ensure fair comparison. As part of future work, deployment trade-offs will be profiled empirically—latency, memory footprint, and energy consumption—across backbone scales and adapter configurations to quantify the cost–accuracy balance.

## Figures and Tables

**Table 1:** Raymobtime-s007: metadata and channel-path fields.

File	Field	Units	Description
CSV	EpisodeID	–	Simulation episode (0–49)
CSV	SceneID	–	Temporal scene inside episode (0–39)
CSV	VehicleArrayID	–	Receiver index (0–9)
CSV	VehicleName	–	SUMO vehicle tag
CSV	x,y,z	m	3D receiver coordinates
CSV	Val	V/I	Valid/invalid link flag
CSV	LOS	0/1	1 = line-of-sight to TX
HDF5	Power	dBm	Received power of path
HDF5	ToA	s	Time of arrival
HDF5	AoD (el,az)	°	Angles of departure
HDF5	AoA (el,az)	°	Angles of arrival
HDF5	LOS flag	0/1	1 = path is LOS
HDF5	Phase	°	Ray phase (unused)

**Table 2:** Episode-level split used in this work.

	Train (e0–e39)	Val (e40–e44)	Test (e45–e49)
2.8 GHz	7316	870	892
60 GHz	7316	870	892

**Table 3:** Localization accuracy on the validation and test sets.

Freq.	Validation (m)				Test (m)			
	Mean	Median	RMSE	90th	Mean	Median	RMSE	90th
2.8 GHz	8.746	5.899	14.344	15.555	12.971	8.882	25.805	21.989
60 GHz	4.718	3.249	7.206	8.477	8.154	5.630	12.360	15.719
2.8 GHz→60 GHz	N/A				58.208	44.414	72.051	108.468
60 GHz→2.8 GHz	N/A				101.776	105.748	116.924	168.088

**Table 4:** Comparison of localization error (in meters) between baseline models and the proposed method on the validation and test sets. Results are presented for both 2.8 GHz and 60 GHz frequency bands. The best-performing results from the proposed model are highlighted in bold.

Model	Validation				Test			
	Mean	Median	RMSE	90th	Mean	Median	RMSE	90th
**2.8 GHz**								
MLP	16.81	12.57	22.66	33.08	19.86	14.21	30.06	40.08
GNN	13.36	9.05	19.37	27.09	16.30	10.16	27.38	32.99
CNN	15.58	11.44	20.26	32.66	17.96	12.50	25.29	33.93
kNN	14.71	4.19	28.41	42.90	20.35	5.83	38.55	63.56
**Proposed Model**	**8.75**	**5.90**	**14.34**	**15.56**	**12.97**	**8.88**	**25.81**	**21.99**
**60 GHz**								
MLP	8.38	5.73	11.50	17.31	9.26	6.80	13.18	17.69
GNN	7.26	5.26	10.44	14.10	8.21	5.54	12.94	15.52
CNN	9.40	7.92	10.99	16.06	10.22	8.34	12.16	18.02
kNN	5.39	1.34	13.21	15.47	6.39	1.49	15.53	18.31
**Proposed Model**	**4.72**	**3.25**	**7.21**	**8.48**	**8.15**	**5.63**	**12.36**	**15.72**

**Table 5:** Few-shot localization accuracy (m) and improvement (% with regard to k=0). The ↓ symbol indicates that lower values are better, while the ↑ symbol indicates that higher values are better.

Freq	*k*	Mean ↓	Median ↓	RMSE ↓	ΔMean (%) ↑	ΔMedian (%) ↑	ΔRMSE (%) ↑
2.8 GHz	0	24.70	13.09	53.06	–	–	–
	1	8.75	5.90	14.34	−64.59	−54.93	−72.97
	2	11.38	8.64	15.60	−53.91	−34.01	−70.61
	3	15.25	11.23	22.56	−38.24	−14.18	−57.47
	4	11.06	7.82	16.73	−55.20	−40.29	−68.46
	5	12.28	9.41	16.66	−50.29	−28.06	−68.60
60 GHz	0	10.01	6.91	13.85	–	–	–
	1	4.72	3.25	7.02	−52.88	−52.97	−49.32
	2	4.99	3.47	7.36	−50.18	−49.82	−46.86
	3	6.21	4.44	8.32	−37.95	−35.72	−39.95
	4	6.41	4.35	8.82	−35.98	−37.10	−36.35
	5	7.30	5.51	9.29	−27.12	−20.20	−32.91

**Table 6:** Best-epoch localization statistics for each training data ratio.

Band	Ratio	Mean (m)	Median (m)	$P_{90}$ (m)	Max (m)
2.8 GHz	0.2	16.77	9.92	33.93	182.46
	0.4	12.08	7.80	21.26	146.41
	0.6	10.30	7.09	18.18	142.75
	0.8	9.57	6.42	18.50	157.89
	1.0	8.75	5.90	14.34	170.22
60 GHz	0.2	8.19	5.94	15.17	177.36
	0.4	6.54	4.31	11.89	70.98
	0.6	5.65	4.23	10.73	75.07
	0.8	5.51	4.19	10.06	77.44
	1.0	4.72	3.25	8.48	90.67

**Table 7:** Raw localization errors for every depth configuration on ${\mathrm{fr}}_{1}=2.8$ GHz and ${\mathrm{fr}}_{2}=60$ GHz.

	2.8 GHz				60 GHz			
Layer_Index	Mean	Median	RMSE	$p_{90}$	Mean	Median	RMSE	$p_{90}$
2*0_0*0_0	9.700	6.933	14.060	16.774	5.671	4.363	7.641	10.401
4*0_0*0_0	8.940	6.600	13.496	15.642	5.749	3.915	8.026	11.153
6*0_0*0_0	9.769	7.457	14.646	16.598	6.033	4.476	7.709	11.281
2*0_4*0_4	9.700	6.933	14.060	16.774	5.671	4.363	7.641	10.401
4*0_4*0_4	8.756	5.902	14.340	17.665	5.493	4.140	7.541	10.131
6*0_4*0_4	9.389	6.409	13.843	17.339	5.697	4.446	7.684	11.134
2*0_1_4*0_1_4	9.575	7.210	14.367	16.979	5.955	4.484	8.321	10.716
4*0_1_4*0_1_4	10.268	8.099	14.253	18.779	6.006	4.536	7.797	11.875
6*0_1_4*0_1_4	9.678	7.283	13.937	19.387	5.897	4.477	7.759	10.931
2*0_2_4*0_2_4	9.718	7.170	14.834	17.155	5.031	3.411	7.121	9.800
4*0_2_4*0_2_4	10.492	8.045	15.172	19.251	4.718	3.249	7.206	8.477
6*0_2_4*0_2_4	10.370	8.205	14.916	17.638	5.883	4.554	7.997	10.949

## Data Availability

The dataset used in this work is a publicly available dataset.
